# Characterisation of a Leaky Splice-Site Mutation Associated with Phenotypic Diversity in Two Unrelated Patients with ARPC1B Deficiency

**DOI:** 10.1007/s10875-026-02002-4

**Published:** 2026-03-07

**Authors:** Alex Quach, Jovanka King, Trishni Putty, Michael Gold, Patrick Quinn, Antonio Ferrante

**Affiliations:** 1 Department of Immunopathology, SA Pathology, Women’s and Children’s Hospital, North Adelaide, Adelaide, South Australia Australia; 2 School of Biomedicine, Adelaide Medical School, Robinson Research Institute, Adelaide University, Adelaide, South Australia Australia; 3 The Discipline of Paediatrics, School of Medicine, Department of Allergy and Clinical Immunology, Adelaide University, Women’s and Children’s Health Network, North Adelaide, South Australia Australia; 4 School of Biological Sciences, Adelaide University, Adelaide, South Australia Australia

**Keywords:** Arp2/3 complex, ARPC1B, Immunodeficiency, Inborn error, Leaky splicing

## Abstract

Autosomal-recessive pathogenic variants in *Actin-related protein 2/3 complex subunit 1B* (*ARPC1B*) result in an inborn error of immunity associated with eczema, thrombocytopenia, leukocytoclastic vasculitis and colitis. ARPC1B deficiency leads to impaired actin filament branching, affecting cytoskeletal processes in leukocytes, including migration, adhesion, endocytosis, and phagocytosis. This report presents two index cases from different families, who despite sharing an identical germline homozygous *ARPC1B* splice-site variant, ARPC1B^c.64+2T> A^, had distinct clinical phenotypes including age of onset of symptoms and clinical manifestations. In addition, differences were observed in their immunological profiles including neutrophil chemotaxis, upregulation of complement receptor expression as well as B cell maturation and responses to EBV transformation. Further molecular investigation suggests the downstream cryptic splice-site activated by the mutation permits ‘leaky splicing’, enabling trace expression of wildtype ARPC1B. This supports a less severe deficiency in ARPC1B activity, rendering a less severe phenotype in one case compared with the other due to differential transcriptional activity of wildtype ARPC1B.

**Supplementary Information:**

The online version contains supplementary material available at 10.1007/s10875-026-02002-4.

## Introduction

The actin cytoskeleton is indispensable for the physiological function of eukaryotic cells. It orchestrates a myriad of fundamental cellular processes through its remarkable dynamics and structural plasticity. Composed of filamentous (F)-actin, it undergoes constant assembly and disassembly. Disruption of this system underlies inborn errors of immunity such as Wiskott-Aldrich syndrome (WAS) and ARPC1B deficiency, both marked by defective actin filament formation [1, 2].

Central to actin polymerisation and branching is the actin-related protein (Arp) 2/3 complex, which nucleates new (“daughter”) filaments at 70° angles from existing (“mother”) filaments upon activation by nucleation-promoting factors like the WAS protein family [3–5]. This branching network provides structural support and motility forces for cellular protrusions. The complex includes seven subunits: Arp2 and Arp3, which nucleate F-actin, and regulatory subunits, Arp2/3 complex (ARPC) 1, 2, 3, 4, and 5 [3, 6, 7]. ARPC1, a β-propeller protein with seven blades, links the Arp2/3 complex to actin filaments [8]. Existing as two isoforms, ARPC1B predominates ARPC1A in hematopoietic cells and has a higher capacity to assemble stable actin branches [7].

In patients with ARPC1B deficiency, hematopoietic cells exhibit a profound disruption in Arp2/3-mediated branched actin networks. Consequently, these cells fail to form lamellipodia [9], densely branched actin networks that drive membrane protrusion and cell motility, impairing immune cellular processes including chemotaxis [2], formation of the immune synapse [10], phagocytosis [11], and cell adhesion and spreading [12]. Autosomal-recessive mutations in the *ARPC1B* gene were first reported in 2017, associated with a combined immunodeficiency that resembles WAS [1, 2]. The disease is attributed to at least 20 unique pathogenic variants in the *ARPC1B* gene across over 40 patients, includes splice-site substitutions, missense and nonsense substitutions, duplications and indels causing frameshifts. These occur across the gene with no apparent hotspot, suggesting a sensitivity to gene variation. Clinical features include fever, bloody diarrhoea, atopic manifestations (eczema, food allergy, asthma), recurrent infections (cutaneous, ear, pulmonary), leukocytoclastic vasculitis, colitis, lymphadenopathy, and failure to thrive. Laboratory findings tend to be remarkable for variable hypergammaglobulinaemia, eosinophilia, T cell lymphopaenia, B cell lymphocytosis, morphological, functional and numerical aberration of platelets, along with perturbed cytoskeletal activity, and a functional deficiency in T and NK cells [13, 14].

We report two cases of ARPC1B deficiency in unrelated kindreds, carrying an identical homozygous c.64 + 2T > A mutation in *ARPC1B* gene (NM_005720.4). These were previously reported as part of a case series by Volpi et al. [15] along with a United States-based kindred, and we independently verified the mutation by Sanger sequencing. The same variant was recently reported in a cohort of 14 Nepalese patients in which a founder effect occurred [16]. In our proband duo, we noted a disparity in the severity of the clinical phenotype, with one patient requiring urgent haematopoietic stem cell transplantation (HSCT) and the other with evolving clinical features over time. This prompted further investigation into clinical, functional, and laboratory differences to explore underlying mechanisms.

## Methods

### Patients

The procurement of human blood and all experimental procedures were approved by the Human Research Ethics Committee of the Women’s and Children’s Health Network (WCHN), Adelaide, South Australia, in accordance with The National Statement on Ethical Conduct in Human Research (2007, updated 2018; National Health and Medical Research Council Act 1992). Blood was collected from patients, their parents, and unrelated healthy volunteers by venepuncture, with their informed consent. Results from studies on patients and their parents were obtained as part of the clinical laboratory assessment of the patient and informed consent was obtained from the parents to publish the results. This study was conducted in accordance with the Helsinki Declaration.

### Reagents

RPMI 1640 medium supplemented with L-glutamine was purchased from Invitrogen. Penicillin-streptomycin solution, Medium 199, bovine serum albumin (BSA) and Formyl-Met-Leu-Phe (fMLF) was purchased from Sigma-Aldrich. Human recombinant tumour necrosis factor (TNF) was purchased from ProSpec. Foetal bovine serum (FBS) was purchased from Serana Australia. FBS was heat-inactivated (HI) by incubation at 56 °C for 30 min. Antibodies used throughout this study are described in Table S1.

### Isolation of Peripheral Blood Mononuclear Cells and Neutrophils

Leukocytes were isolated from the blood of healthy donors using the rapid-single-step method, as described previously [17]. The two leukocyte fractions, mononuclear (PBMC) and polymorphonuclear (PMN, neutrophils) cells, were harvested separately and washed and either used immediately or archived by pelleting in aliquots at 2 × 10^6^ per vial and stored in the absence of media at -80 °C.

### Cell Lines

EBV-transformed lymphoblastic cell lines (LCLs) were established from B cells of patients and their parents, as well as unrelated healthy donors, using established methods [18, 19]. K562, a human erythroid myeloid cell line, was kindly provided by Dr. Geoff Shellam, Department of Microbiology, University of Western Australia. All cell lines were maintained in RPMI-10% HI FBS at 37 °C in a humidified 5% CO_2_ incubator.

### Lymphocyte Immunophenotyping

Immunophenotyping of lymphocyte subsets was performed on blood using a fluorescent-conjugated antibody cocktail (comprised of anti-human CD45 APC-H7, CD3 FITC, CD16 PE, CD56 PE, CD19 PE-Cy5, CD4 APC, and CD8 PE-Cy7) as previously described [20].

### Natural Killer Cell Cytotoxicity

NK cytotoxicity assay was assessed using carboxyfluorescein succinimidyl ester (CFSE)-labelled K562 target cells co-incubated with NK cell-containing PBMC at effector: target (E: T) ratios 1:1, 2:1, 5:1, 10:1, 25:1, 50:1, and 100:1 for 4 h at 37 °C in a humidified 5% CO_2_ incubator. After incubation, the cells were stained with 7-aminoactinomycin D (7-AAD) and acquired on a FACSCanto flow cytometer. The percentage of CFSE^+^(7-AAD)^+^ cells (lytic K562) was determined for each E: T relative to a K562-absent and total lysis controls, and assessed against the absolute NK count in each E: T, which was determined relative to immunophenotyping and complete blood enumeration. The lethal dose of NK cells for 25% lysis of K562 (LD25) was interpolated from a non-linear least squares-fitted curve of the NK count per E: T versus lytic K562%.

### Neutrophil Function

Neutrophil chemotaxis was measured using the under-agarose method, slightly modified from previously described [21, 22]. Medium 199 (2.2X) supplemented with sodium bicarbonate and 10% HI FBS was mixed with molten agarose (2%) and set in 60 × 15 mm tissue culture plates (Corning). Triplicate 2.5 mm diameter wells were cut 3 mm apart, with 5 µL of 4 × 10^6^ neutrophils added to the central well, and 5 µL of 10^− 7^ M fMLF or DMSO added to adjacent wells. After 90 min at 37 °C in a humidified 5% CO_2_ incubator, migration towards the fMLF well was measured under an inverted Olympus CKX41 microscope.

Neutrophil bactericidal activity against *Staphylococcus aureus* (NCTC 6571, Oxford strain) was measured by colony counting similarly to previously described [23]. Briefly, 1 × 10^6^ neutrophils were incubated with 1 × 10^6^ *S. aureus* in 500 µL HBSS supplemented with 10% human AB serum in 5% CO_2_ infused 5 mL polypropylene tubes on a rocking platform at 37 °C. Samples were taken at 0 and 2 h, diluted 1/200 in sterile water, and 50 µL aliquots were plated on horse blood agar. After incubation at 37 °C overnight, colonies were counted, with bacterial killing % calculated as [(0 h colonies − 2 h colonies) / 0 h colonies] × 100.

Neutrophil complement receptor immunoglobulin (CRIg) and CD11b upregulation was assessed in whole blood as previously described [24]. Fifty µL of whole blood was incubated with 10^− 6^ M fMLF or TNF 10^3^ U/mL for 20 min, followed by staining with fluorochrome-conjugated anti-human CRIg or CD11b and CD45 antibodies for 15 min. Erythrocytes were lysed and leukocytes were fixed by incubating with BD FACS Lysing solution for 10 min. Following washing with PBS, the cells were acquired on a BD FACSCanto and analysed using FlowJo 10.8. Stimulation indices were calculated as the ΔMFI (isotype control subtracted) divided by the corresponding ΔMFI of unstimulated neutrophils.

### Actin Polymerisation

LCLs were stained for F-actin using phalloidin and the BD Cytofix/Cytoperm kit. Cells (5 × 10^6^/mL) were fixed with 250 µL Fixation/Permeabilization solution for 20 min, washed and resuspended in 500 µL 1x Perm/Wash for 10 min. Following another centrifugation, the supernatant was decanted, and a titrated volume (2.5–10 µL) of 1.5 µM Alexa Fluor™ 488 Phalloidin (Invitrogen) added to the cells, with incubation in the dark for 15 min. After washing, cells were either acquired on a BD FACSCanto flow cytometer and assessed for fluorescence in the FL-1 detector using FlowJo 10.8; or cytospun (100 µL) onto glass slides, for visualisation under oil immersion (100x) on an Olympus BX51 Fluorescence Microscope.

### Western Blot

Cell pellets (2 × 10^6^) were lysed in 100 µL buffer containing 20 mM HEPES, pH 7.4, 0.5% Nonidet P-40 (v/v), 100 mM NaCl, 1 mM EDTA, 2 mM Na_3_VO_4_, 2 mM dithiothreitol, 1 mM phenylmethylsulfonyl fluoride, and cOmplete™, EDTA-free Protease Inhibitor Cocktail (Roche). Total protein was quantified using the Qubit™ Protein Assay Kit on a Qubit 3.0 (Invitrogen). Samples in Laemmli buffer were boiled (100 °C, 5 min), resolved by 10% SDS-PAGE (170 V, 45 min), and transferred to nitrocellulose membranes using the Trans-Blot^®^ Turbo™ Transfer System (Bio-Rad), with total protein examined by 0.1% Ponceau S staining. Membranes were blocked in Tris-buffered saline with Tween-20 (TBST) with either 5% skim milk (or 5% BSA, where indicated), incubated with anti-ARPC1B primary antibodies, washed, then incubated with HRP-conjugated antibodies. Detection was performed using the Clarity™ Western ECL Substrate (Bio-Rad) and visualised on a ChemiDoc™ XRS+ Imager using Image Lab™ Software, Version 3.0 (Bio-Rad). For ARPC1A or GAPDH detection, membranes were stripped with ReBlot Plus Mild Antibody Stripping Solution, washed, and re-probed with respective antibodies.

### ARPC1B Mutation Detection and Transcript Analysis

Genomic DNA was isolated from heparinised blood or 2 × 10^6^ LCLs using the Flexigene DNA kit (Qiagen). Total RNA was extracted from 2 × 10^6^ LCLs using TRIzol (Invitrogen) and converted cDNA from 300 ng RNA using iScript™ cDNA synthesis kit (Bio-Rad). AmpliTaq Gold^®^ 360 Master Mix (Applied Biosystems) was used to amplify: *ARPC1B* exon 2 and the flanking intronic regions from genomic DNA with 0.1 µM M13-tagged primers (Forward: GCTGCCCCTCTAAACTGAGG; Reverse: AACTTTAACCCAGGAGGCCC); ARPC1B mRNA with primers (Forward: 5’-TCGACTGCCCAGAGTCCG-3’ on exon 1; Reverse: 5’-TGCAAACCCACCAGTCATTC-3’ overlapping exon 4–5 junction). Thermal cycling conditions were 5 min at 95 °C followed by 35 cycles at 95 °C for 30 s, 60 °C for 30 s and 72 °C for 30 s. PCR products were visualised on a 2% agarose gel, purified using Illustra ExoProStar 1-Step (GE HealthCare) and sequenced using BigDye Terminator v3.1 on an ABI 3730 DNA Analyzer (Applied Biosystems). Sequences were aligned to reference sequences NG_055676.1 (genomic DNA) and NM_005720.4 (cDNA) with Mutation Surveyor v5.0.1 (SoftGenetics, State College, PA).

### Quantitative Allele-Specific PCR for ARPC1B Wildtype and Mutant Transcripts

Quantitative allele-specific amplification of the wild-type or ARPC1B^c.64+2T> A^ transcripts was performed using a Bio-Rad iQ5 Real-time Detection System. Primer designs and combination usage are presented in Table S2 and were from Bioneer Pacific. Each 12.5 µL reaction contained 6.25 µL SsoAdvanced Universal SYBR^®^ Green Supermix (Bio-Rad), 100 nM amplification primers, 200 nM blocking primer where applicable, and 0.5 µL cDNA. *ARPC1A* and *GAPDH* (housekeeping control) were amplified without blocking primers. Thermal cycling conditions were 95 °C for 30 s followed by 40 cycles of 95 °C denaturation for 10 s and an annealing/extension step of 30 s at 60 °C. Melting curve analysis confirmed product specificity. Relative expression was determined using the ΔΔCt method.

### Statistics

RStudio 2023.12.1 Build 402 [25] with R 4.3.3 [26] was used for statistical analysis. R packages used included ggplot2 [27], ggpubr [28], rstatix [29], ggstatsplot [30] and emmeans [31]. Mean differences were compared using t-tests (for comparisons of two groups) or one-way ANOVA followed by multiple-comparison tests (for comparisons of three of more groups). *P* values < 0.05 were considered statistically significant.

## Results

### Clinical Trajectory of Two Index Patients with the Homozygous ARPC1B^c.64+2T> A^ Mutation

**Fig. 1:**
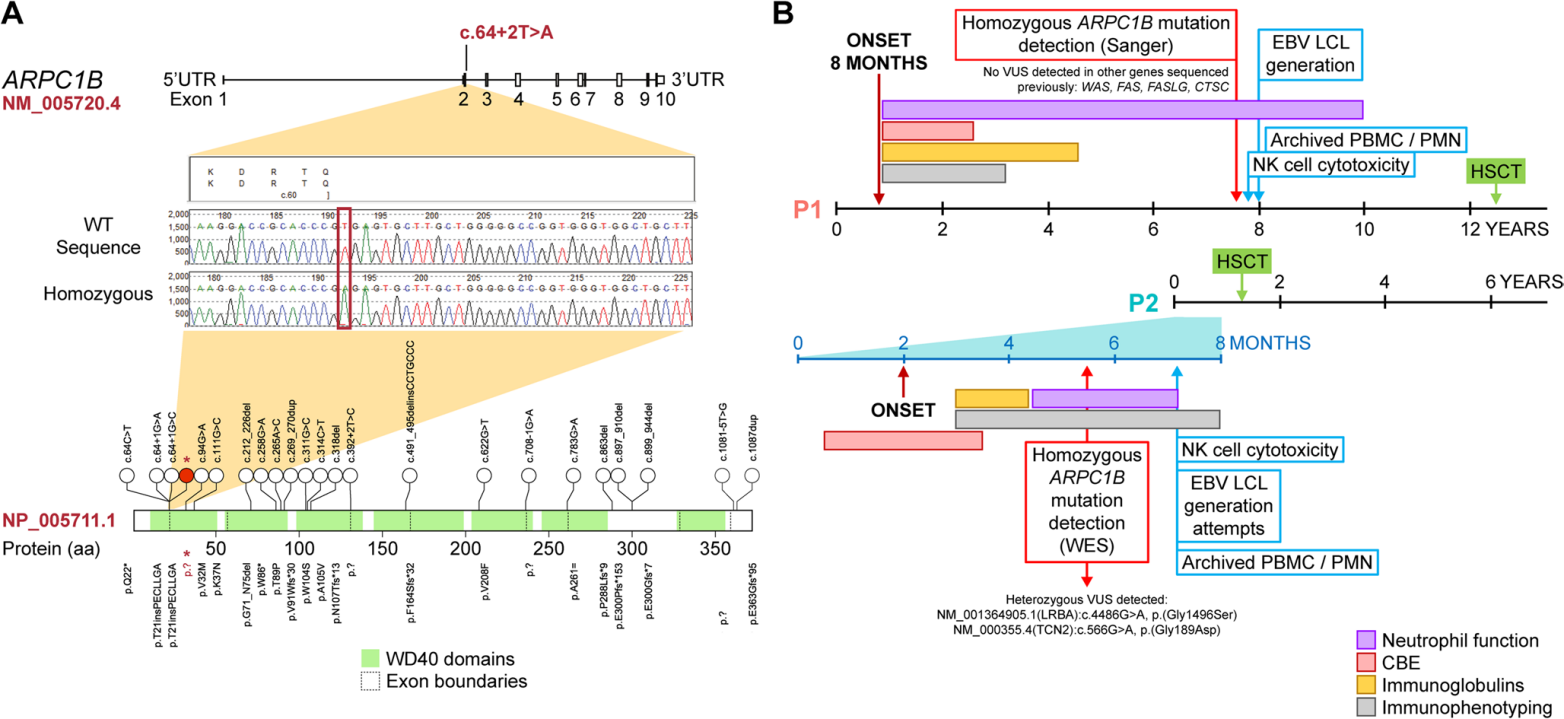
The ARPC1B ^c.64+2T> A^ mutation identified in two ARPC1B deficiency cases and their laboratory investigation timeline. (**A**) The germline homozygous ARPC1B^c.64+2T> A^ mutation detected in P1 and P2. The variant is an intronic single nucleotide substitution at position + 2 of the last base of exon 2. The locations of other reported pathogenic variants are mapped along ARPC1B. These are tabulated in Table S3. aa, amino acids. (**B**) Relative timelines of the laboratory investigation in the diagnosis and characterisation of P1 and P2. *LCL*, lymphoblastoid cell line; *CBE*, complete blood enumeration

We investigated two unrelated patients both carrying a homozygous ARPC1B^c.64+2T> A^ substitution (Fig. 1A, Table S3). The probands, a male (Patient 1, P1) and a female (Patient 2, P2), were born to non-consanguineous parents of Nepalese heritage. They were reported as ‘P14’ and ‘P12’ in the ARPC1B patient series reported by Volpi et al., respectively [15]. Clinical and laboratory features for both probands are summarised in Table 1.

**Table 1:** Clinical manifestations of ARPC1B-deficient patients with homozygous ARPC1B^c.64+2T> A^ mutations

Clinical characteristic	P1	P2
Sex	Male	Female
Ethnicity	Nepalese (family from Bhutan)	Nepalese
Consanguinity	No	No
Disease onset	8 months of age	2 months of age
Haematologic	• Microcytic anaemia • Thrombocytopaenia • Hypereosinophilia	• Microcytic anaemia • Thrombocytopaenia • Hypereosinophilia
Infections	• Recurrent otitis media • Chronic bronchitis • Severe molluscum contagiosum • Severe, early onset periodontal disease with loss of bone – complete extraction of primary dentition aged 5 years	• Recurrent UTI (*Enterobacter* spp.) • Periorbital cellulitis • Oral candidiasis • Recurrent suppurative otitis media • CMV (postnatal acquisition), adenovirus, rotavirus (vaccine strain) • Ulcerative lesions: perianal (*Pseudomonas* spp.), eyelid, gum, vulva, lip, ear (*Candida albicans*, MSSA)
Inflammatory	• Polyarthritis • Chronically elevated inflammatory markers • Non-specific granulomatous inflammation (lymph node biopsy) • Marked lymphadenopathy in infancy	• Cutaneous vasculitis with perivascular inflammation • Granulomatous lung lesion (right upper lobe)
Cutaneous	• Mild eczema	• Erosive dermatitis with purpuric and eczematous areas • Recurrent MSSA skin infections • Leukocytoclastic vasculitis on the scalp • Spongiotic dermatitis • Poor wound healing
Gastrointestinal	• Protracted diarrhoea • Short stature / Failure to thrive	• Diarrhoea, cow’s milk protein allergic enteritis with severe metabolic acidosis • Transient hepatitis
Respiratory	• Chronic cough • Bilateral bronchiectasis	• Cystic pulmonary lesion of unclear aetiology – likely granulomatous
HSCT	12 years of age	2 years of age

P1 had a milder, chronic, and more slowly evolving disease. He first presented at 8 months with significant lymphadenopathy, later developing severe periodontal disease with early dental exfoliation, chronic diarrhoea, eczema, chronic wet cough, and progressive bronchiectasis and polyarthritis. Genetic investigation was prompted when a loss of heterozygosity region was detected on a CGH microarray, leading to Sanger sequencing, which confirmed the same ARPC1B variant as P2. Due to progressive disease, he underwent HSCT at 12 years of age.

In contrast, P2 exhibited a more severe phenotype, presenting at 2 months of age with severe bloody diarrhoea, failure to thrive, cutaneous vasculitis, and recurrent polymicrobial infections (further details in Table 1). Due to concern for a severe IEI, urgent whole-exome sequencing (WES) identified the newly described ARPC1B mutation. In addition to the homozygous ARPC1B c.64 + 2T > A variant, P2 harboured heterozygous variants of uncertain significance (VUS) in *LRBA* and *TCN2* (Fig. 1B). Both variants were monoallelic, and neither gene is known to cause disease in the heterozygous state. No further analysis of these genes was permitted within the diagnostic scope under which testing was performed. The severity of her condition necessitated HSCT at 2 years of age, which was successful, and she remains well after 7 years of follow-up.

A timeline of diagnostic testing for both patients is presented in Fig. 1B.

### Laboratory Investigations During Early Disease Onset and Development

**Fig. 2:**
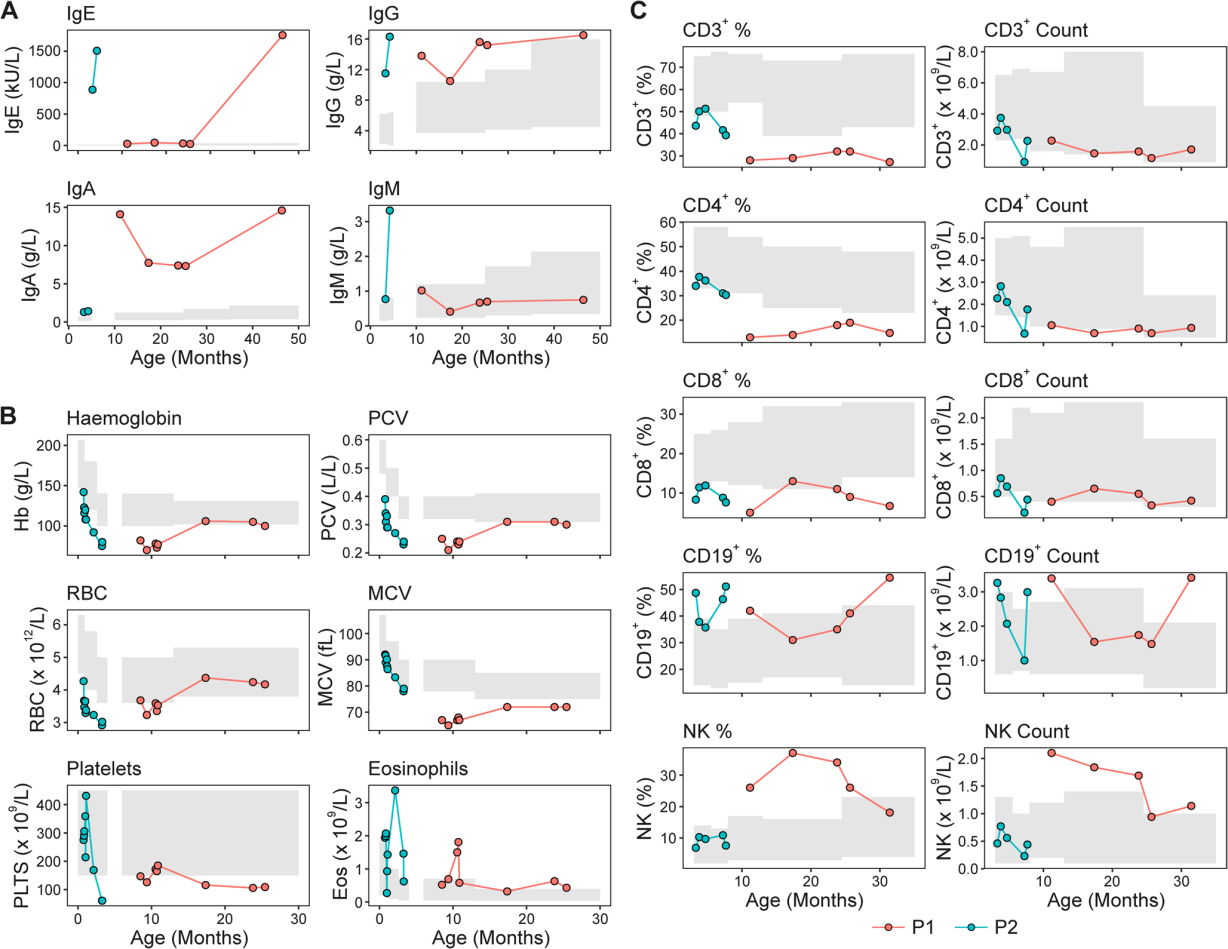
Routine laboratory immunological blood parameters during initial period of disease presentation for P1 and P2. (**A**) Immunoglobulin levels. (**B**) Red blood cell, platelet and eosinophil parameters. (**C**) Lymphocyte immunophenotyping proportions and counts. The grey shaded regions indicate the age-related normal intervals

Given the disparity in the clinical phenotype of P1 and P2, a retrospective examination of their pathology laboratory testing records was undertaken to determine whether a similar difference would be detected during early disease onset and development. Similarities were revealed between P1 (first 4 years) and P2 (first 8 months). Both exhibited elevated immunoglobulin levels, particularly IgE, IgG and IgA (Fig. 2A), with P1 showing a dramatic IgE was increase over time. Routine complement testing was normal in both (C3, C4, CH50, see Table S4). Both had microcytic anaemia, with evolving thrombocytopaenia with normal platelet volume and eosinophilia (Fig. 2B), consistent with other ARPC1B deficiency cases. P1 demonstrated an impaired serological response to polysaccharide pneumococcal vaccination (Pneumovax 23). Lymphocyte subset analysis revealed a mild T cell lymphopaenia (Fig. 2C). P1 had fluctuating B cell counts, and elevated NK cell counts. Despite the T cell lymphopaenia, both had normal proliferation responses to PHA and other stimuli (Table S4). Anti-CD3 stimulated proliferation was only tested for P2, but was found to be normal.

### Ex Vivo Cellular Function Investigations Demonstrate Phenotype Disparity

Advanced functional tests revealed actin cytoskeletal activity differences between P1 and P2 that paralleled their clinical severity.

**Fig. 3:**
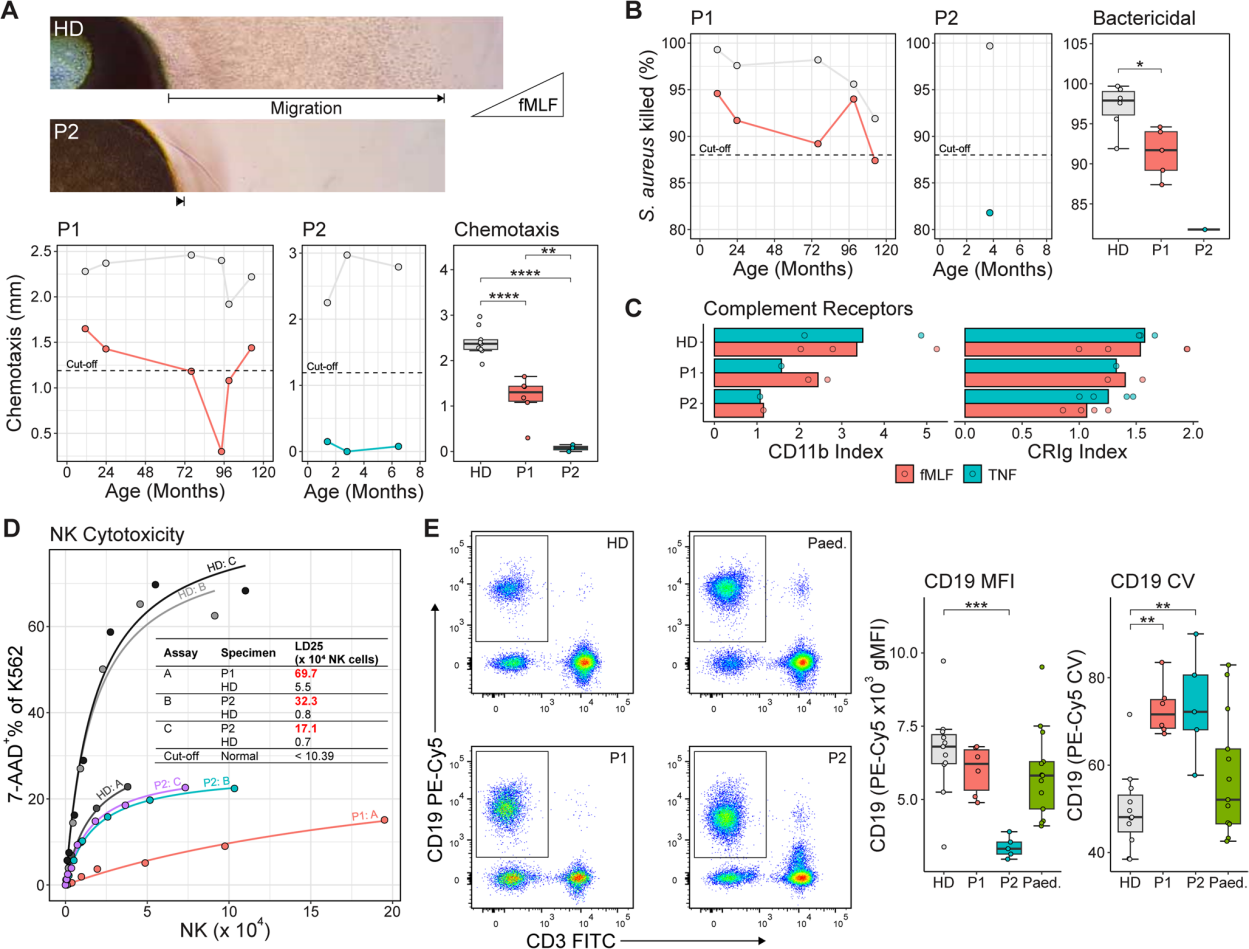
In vitro immune function assessments. (**A**) Neutrophil chemotaxis serial measurements and boxplot comparison. Inset are representative brightfield microscopy images of neutrophils migrating through agarose towards an fMLF loaded well (outside of the images). (**B**) *Staphylococcus aureus* killing by neutrophils. Serial measurements and boxplot comparison. (**C**) Complement receptor upregulation index for CD11b and CRIg. P2 data is from the same source as reported in Small et al. [24] (**D**) Natural killer cell cytotoxicity curves. Inset table shows NK cells to achieve a lethal dose of 25% cytotoxicity (LD25) for each specimen; low cytotoxicity results (above laboratory LD25 cut-off) are indicated in red text. (**E**) CD19 expression levels from retrospective immunophenotyping analysis. Representative flow cytometric dot plots are shown alongside boxplot comparisons of PE-Cy5 mean fluorescence intensities (MFI) and coefficient of variation (CV) of the CD19 + population. Bonferroni-corrected multiple comparisons testing: * *P* < 0.05, ** *P* < 0.01, *** *P* < 0.001, **** *P* < 0.0001. For all boxplots: the boxes represent the interquartile range (IQR; 25th to 75th percentile), with the line inside each box indicating the median (50th percentile). Whiskers extend to the smallest and largest values within 1.5 times the IQR. Points beyond the whiskers represent outliers

Neutrophil chemotaxis was assessed using an in-house under-agarose method with an fMLF gradient (Fig. 3A). P1 underwent six assessments over nearly a decade, consistently demonstrating reduced but borderline-normal chemotaxis (mean = 1.18 mm; normal cutoff = 1.19 mm) compared to healthy donors (HDs) (mean = 2.41 mm). while P2, assessed three times shortly after disease onset, exhibited minimal migration (mean = 0.08 mm). Statistically, P1’s chemotaxis was significantly reduced from HDs (*P* < 0.0001), and P2’s was significantly lower than P1’s (*P* < 0.01). These findings suggest partial ARPC1B function in P1, enabling limited migration via partial lamellipodia formation.

Neutrophil respiratory burst activity was normal in both patients (dihydrorhodamine-123 assay, Table S4), but bacterial killing capacity against *S. aureus* was slightly reduced (Fig. 3B). P1 showed a consistent reduction (mean = 91.4% *S. aureus* killing) compared with HDs (mean = 97.1%), whereas killing by P2’s neutrophils was also found to be reduced (81.8%, one measurement, normal cutoff = 88%), implying impaired neutrophil phagocytic efficiency. Neutrophil surface upregulation of complement receptors, CD11b and complement receptor immunoglobulin (CRIg) in response to fMLF and TNF (Fig. 3C), revealed intermediate responses in P1 but marginal increases in P2, consistent with partial cytoskeletal functionality in vesicular transport of newly synthesised receptors to the cell surface.

NK cell cytotoxicity tested against K562 target cells (Fig. 3D). Although the assays were performed on different occasions, both patients consistently showed reduced cytotoxic capacity compared to respective HDs. This reflects a lack of lamellipodia formation, impairing target interaction via the immunological synapse.

Retrospective assessment of CD19 expression from lymphocyte subset immunophenotyping data revealed lower CD19 levels in P2’s B cells compared to P1 and HDs, with both patients showed greater variability (CV) in expression (Fig. 3E). While our data do not allow definitive assignment of B cell maturation subsets, altered CD19 profiles in ARPC1B deficiency have been previously reported [15, 32]. These abnormalities are thought to reflect impaired cytoskeletal regulation of BCR diffusion, thereby skewing the threshold for tonic BCR signalling. The more pronounced reduction in median CD19 expression in P2 may indicate a relative shift toward less mature B cell populations compared to P1, although this remains speculative.

**Fig. 4:**
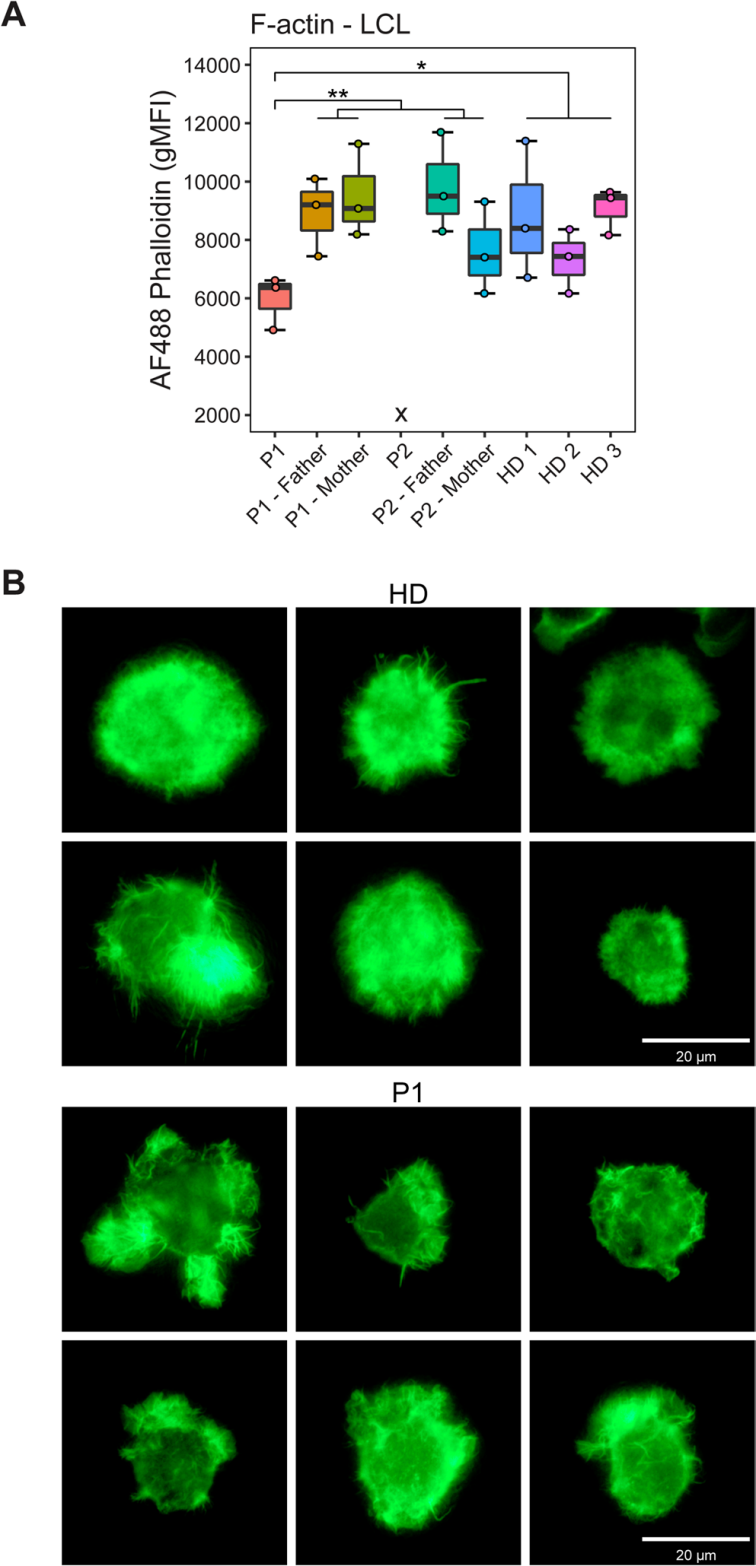
F-actin polymerisation. (**A**) Comparison of geometric mean fluorescence intensities from Alexa-Fluor 488 Phalloidin staining of LCLs from P1, heterozygotes for ARPC1B^c.64+2T> A^, and three healthy donors. Note that P2 was unable to be assessed due to EBV-transformation failure on two attempts. (**B**) Fluorescence microscopic images of LCL from a representative healthy donor and P1. Note the increased prominence of filopodia in P1 LCLs. Bonferroni-corrected multiple comparisons testing: * *P* < 0.05, ** *P* < 0.01

To facilitate further investigations, B cells from the patients and their parents were subjected to an Epstein-Barr virus (EBV) transformation protocol to generate immortalised lymphoblastoid cell lines (LCLs). Transformation succeeded for P1 and both parents but failed for P2, despite two attempts. This contrasts with prior reports, where EBV-transformation difficulties in ARPC1B-deficient B cells were undocumented. P1 LCLs showed reduced F-actin levels compared to heterozygotes and HDs, who exhibited similar levels (Fig. 4A). Fluorescent microscopy revealed extensive filopodia on phalloidin-stained P1 LCLs, indicating a preference for filopodia over lamellipodia formation, consistent with impaired actin branching in ARPC1B deficiency (Fig. 4B).

### Detection of Residual ARPC1B Expression

**Fig. 5:**
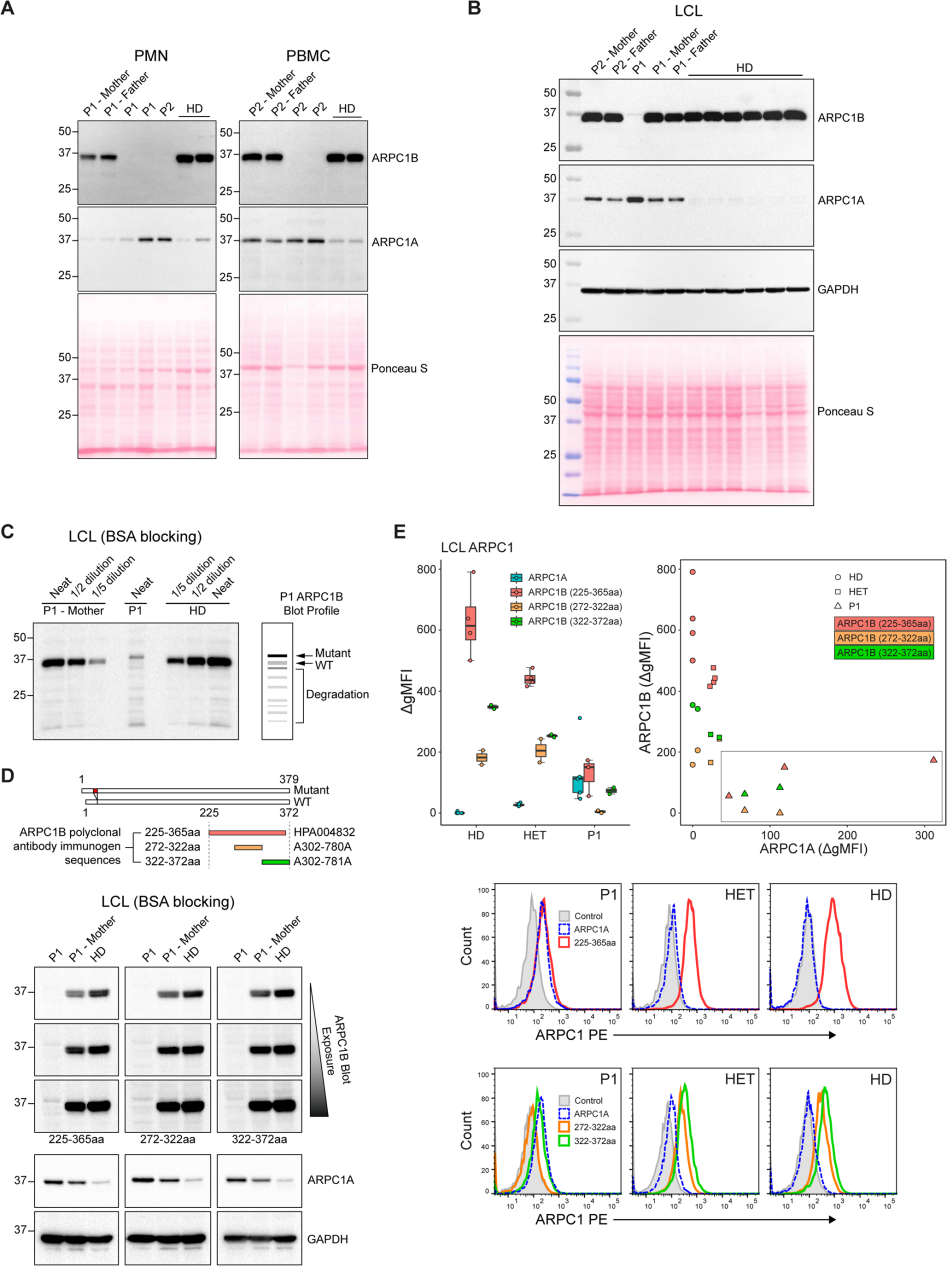
ARPC1 isoform expression. (**A**) Western blotting of ARPC1B/A expression in PMN and PBMC. Note GAPDH expression was not available for these blots. (**B-D**) Western blotting of ARPC1B/A expression in LCL. Substituting skim milk for BSA in blocking enhanced detection of ARPC1B, as observed between (**B**) and (**C**-**D**), revealing mutant, WT, and degradation ARPC1B bands in P1 LCLs. Note that the mutant band is not evident in a heterozygote. Polyclonal ARPC1B antibody produced by 225-365aa (HPA004832) immunogen was used in (**A**-**C**), with staining compared with alternate polyclonal antibodies produced with immunogens to ARPC1B 272-322aa and 322-372aa in (**D**). An abstract immunogen sequence alignment relative to the WT/mutant sequences is also shown in (**D**). (**E**) Intracellular flow cytometry of ARPC1B/A expression in LCL. Box and XY plot of ΔgMFI of ARPC1 isoform staining against control staining for HD (*n* = 2–5), heterozygotes (*n* = 2–4), and P1 (*n* = 1, 2–5 replicates). Representative histograms of ARPC1 isoform expression for each genotype are shown in two rows: the top row shows staining by the ARPC1B 225-365aa antibody, whereas the bottom shows staining by the alternate antibodies as described in (D). For all boxplots: the boxes represent the interquartile range (IQR; 25th to 75th percentile), with the line inside each box indicating the median (50th percentile). Whiskers extend to the smallest and largest values within 1.5 times the IQR. Points beyond the whiskers represent outliers. The P1 data cluster is highlighted by a box in the XY plot

The clinical severity of each case aligned with their laboratory phenotypes, suggesting a protective factor in P1. Functional data hinted at residual ARPC1B expression; however, Western blotting of polymorphonuclear and PBMC lysates from both cases did not detect ARPC1B (Fig. 5A). Elevated ARPC1A levels were consistent with the inverse expression relationship in hematopoietic cells as noted by Volpi et al. [15] and confirmed here.

In P1’s LCL lysate, overexposed blots revealed a faint higher molecular weight band consistent with the 7-amino acid (aa) extended mutant ARPC1B (Fig. 5B). When BSA was used as the blocking agent, detection of this band improved and additional low-abundance species were observed, including a faint band at the expected size of the wild-type protein and lower molecular weight products suggestive of additional splicing products, degradation or reduced stability (Fig. 5C). The identity and biological significance of these bands remain uncertain. Dilution of a heterozygote lysate minimised masking effects and showed that mutant ARPC1B, if present, was expressed only at very low levels. Specificity of both the mutant and WT bands was confirmed using alternative polyclonal antibodies raised against distinct C-terminal ARPC1B epitopes (Fig. 5D).

To complement Western blotting and improve sensitivity, an intracellular flow cytometry assay was developed to measure ARPC1 isoforms using the same antibody panel. This assay demonstrated markedly reduced ARPC1B in P1’s LCLs relative to heterozygotes and HDs, with a reciprocal increase in ARPC1A (Fig. 5E). The relative proportions of ARPC1A and ARPC1B in P1 formed a distinctive expression profile compared with HDs, consistent with compensatory upregulation of ARPC1A in the setting of ARPC1B deficiency, which were unable to be compared with P2 due to their unsuccessful B cell transformation. As the assay was developed post-transplant, testing was limited to archived LCLs.

The relative ΔgMFI of intracellular ARPC1 isoform expression in LCLs showed that even in P1, ARPC1A could not reach normal ARPC1B expression levels, reinforcing that ARPC1A, in addition to its lower actin polymerisation capacity, cannot fully compensate for ARPC1B’s function.

### Investigating the Splicing Mechanism of ARPC1B^c.64+2T> A^

**Fig. 6:**
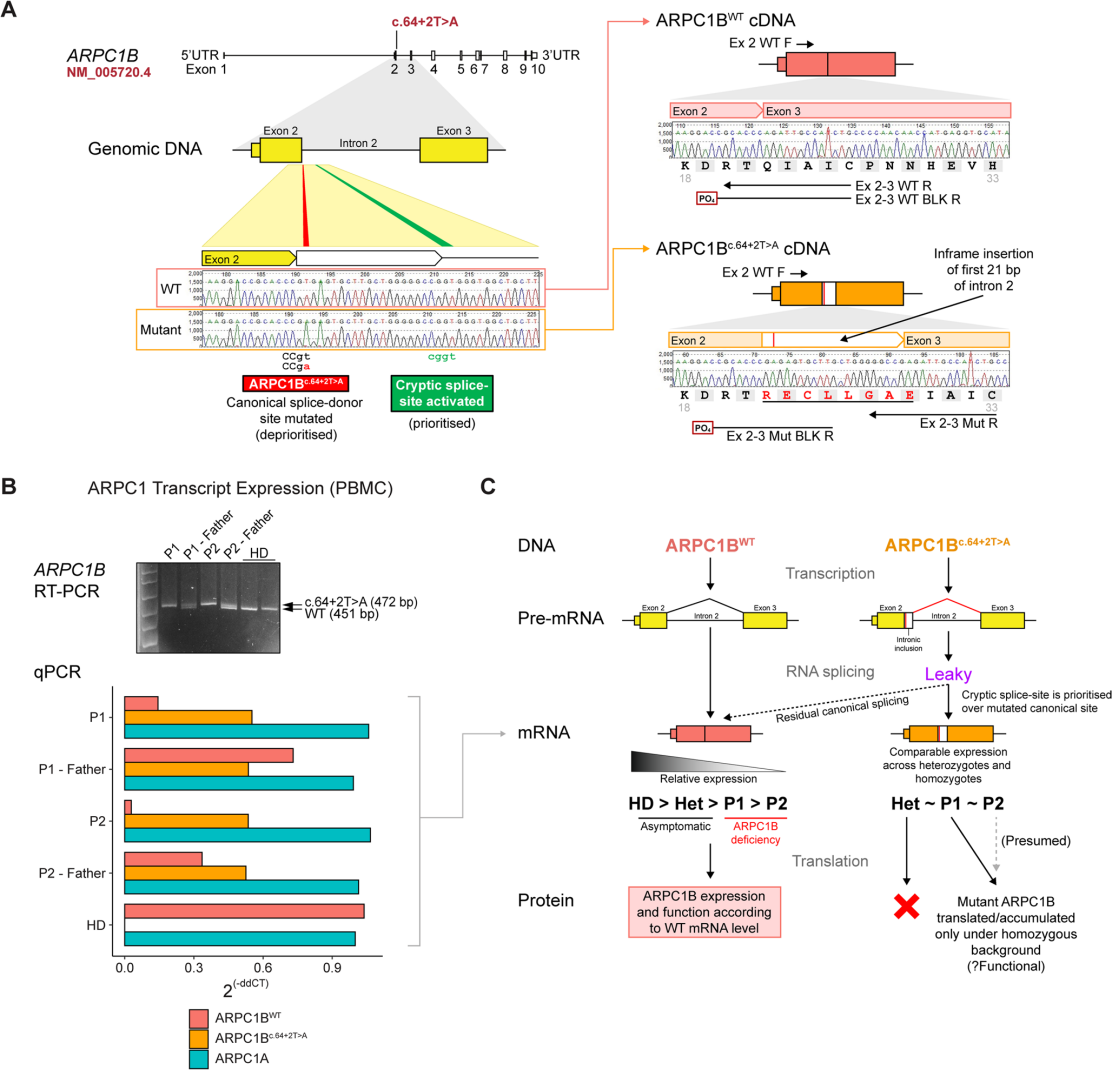
Characterisation of the ARPC1B^c.64+2T> A^ splicing mechanism. (**A**) Splicing mechanism schematic aligned with representative genomic and cDNA transcript Sanger sequencing data of the final wildtype and mutant spliced sequences. The mutant spliced mRNA/cDNA heralds an insertion of the first 21 bp of intron 2 due to activation of a cryptic splice-site. The annealing positions of the allele-specific WT/mutant ARPC1B primers used in the qPCR assay are mapped (refer to Table S2 for the oligonucleotide sequences). The assay included WT or mutant-specific primers for amplification, as well as non-productive phosphate-modified oligonucleotides, designed to anneal to either the WT or mutant transcript to inhibit amplification and enhance specificity of the target amplification. (**B**) Relative ARPC1 isoform transcript/mRNA expression in PBMC. The inset RT-PCR gel electrophoresis image taken of a Gel-Red infused 2.5% agarose with 1 kb Plus DNA Ladder. The same specimens were used in qPCR. HD data is the mean of *n* = 2. (**C**) Schematic flow chart demonstrating the proposed leaky RNA splicing mechanism affecting the ARPC1B^c.61+2T> A^ patients

The impact of the ARPC1B^c.64+2T> A^ mutation on pre-mRNA splicing was uncharacterised although SpliceAI scores and Pangolin per-transcript scores (spliceailookup.broadinstitute.org, Fig. S1) clearly predict the activation of a cryptic downstream splice site (21 bases downstream exon 2, in intron region) [33, 34]. RNA from P1’s LCLs was reverse transcribed to cDNA and sequenced, revealing the insertion of the first 21 bases of intron 2 into the *ARPC1B* mRNA (Fig. 6A). This was due to the loss of the canonical 5’ splice donor site at the exon 2-intron 2 junction and activation of a cryptic downstream splice site. The in-frame insertion predicted a mutant ARPC1B protein with a deletion of Gln22 and insertion of seven additional amino acids (p.Q22delinsRECLLGAE).

This mechanism mirrors that of c.64 + 1G > A and c.64 + 1G > C mutations reported by Brigida et al. [13], which activate the same cryptic splice-site. Unlike those cases, where somatic reversion restored a WT allele, genomic DNA from P1’s blood, PBMCs, and LCLs consistently lacked the WT ARPC1B allele (Fig. S2). Given P1’s chemotactic data and the neutrophil contribution to the DNA pool, leaky splicing more likely could explain the milder phenotype compared to P2.

RT-PCR confirmed higher-molecular-weight mutant *ARPC1B* transcripts in PBMCs from P1, P2, and heterozygotes (Fig. 6B). Allele-specific qPCR showed stable *ARPC1A* expression across all genotypes and absence of the mutant *ARPC1B* transcript in HDs. The mutant transcript was present in both homozygotes and heterozygotes, suggesting evasion of nonsense-mediated decay. WT *ARPC1B* levels correlated with genotype: highest in HDs, intermediate in heterozygotes, and lowest in homozygotes. Notably, the higher WT transcript level observed in P1 (13.9% of HD) compared to P2 (2.7% of HD), suggests a less severe biochemical defect and intermediate cellular responses relative to P2. This pattern of transcript expression corresponded with the protein profile observed on Western blotting. Together, these findings support a model where residual WT splicing in P1 accounts for the phenotypic variability compared with P2 (Fig. 6C).

## Discussion

An in-depth laboratory investigation into the pathological characteristics of an unrelated pair of ARPC1B-deficient patients with the identical homozygous splice-site mutation, ARPC1B^c.64+2T> A^, but disparate clinical phenotype, revealed a corresponding contrast in laboratory cellular function assays. This is conceivably attributable to leaky splicing at the transcriptional level, where the “milder” case, P1, had a detectable level of wild-type *ARPC1B* mRNA that was increased over the “severe” case, P2. It may be inferred that the difference between the patients is due to corresponding levels of wild-type ARPC1B expression and function, permitted by the leaky splicing.

Advancement in genomics and disease knowledge, prompted by the rapid diagnosis required for P2 due to clinical deterioration, facilitated the diagnosis of P1. Functional in vitro assays demonstrated a severely impaired neutrophil migration capacity P2, which corresponded with an actin polymerisation deficiency. In contrast, serial measurements for P1 over several years demonstrated moderately impaired neutrophil chemotactic responses, which intriguingly corresponded with their less severe presentation. The P1/P2 disparity was observed across other immunological parameters, including neutrophil bactericidal activity, complement receptor upregulation and CD19 expression.

Phenotypic variability in ARPC1B deficiency has been described previously, including differences in residual ARPC1B expression, F-actin levels, and podosome formation, even among siblings with the same mutation [1, 32, 35]. In the ARPC1B^A105V^ siblings reported by Volpi et al. [15], variable residual protein was observed, whereas in our cases, variability arises from leaky splicing of ARPC1B^c.64+2T> A^, resulting in differential WT transcript levels. Notably, Volpi et al. also included P1 and P2 in from our study and reported undetectable ARPC1B protein, but did not examine LCLs by intracellular flow cytometry, which in our study revealed trace WT protein in P1 correlating with a milder phenotype. These findings highlight that cellular context and detection method can influence residual ARPC1B measurement and phenotypic interpretation. Although our patients are from separate kindreds, they share ethnicity suggesting a common ancestry, along with a third Nepalese case [15], consistent with ARPC1B^c.64+2T> A^ being a founder mutation.

A study published during revision of this manuscript identified a further 14 patients within 10 Nepalese kindreds homozygous for the same ARPC1B^c.64+2T> A^ splice-site variant, which exemplifies the founder effect and provides important context for our findings [16]. All individuals exhibited severe early-onset disease, although the clinical spectrum was broad and no clearly milder cases were identified. However, the study did not assess residual wild-type ARPC1B transcript or protein expression, quantify isoform-specific ARPC1A/ARPC1B abundance, or examine splice-site usage. As such, potential genotype-independent modifiers – including variation in the efficiency of cryptic splice-site utilisation – could not be evaluated. Our observation that P1 retains a higher proportion of wild-type transcript than P2, despite identical genotype, suggests that variable splice leakage may contribute to phenotypic diversity, although this cannot be confirmed without larger comparative datasets. Interpreting these findings is further complicated by the discordance between transcript and protein abundance: qPCR readily detects low-level transcripts that may not generate stable or measurable protein, particularly in the context of aberrant splicing. ARPC1B was undetectable in PBMCs from both patients by Western blot, consistent with the low abundance and predicted instability of exon 2-skipped or cryptic-splice products. Clarifying the clinical significance of residual transcript and protein expression will require assessment of transcript-protein relationships across additional individuals with the ARPC1B^c.64+2T> A^ variant, ideally using multiple cell types and higher-sensitivity protein detection methods, including in patients from the Nepalese founder population.

The ARPC1B^c.64+2T> A^ variant activates a downstream cryptic splice-site, consistent with other pathogenic variants affecting the canonical donor site of intron 2 [13]. Although somatic reversion has been described in ARPC1B deficiency, the available data in this study more strongly support variable splice-site usage – rather than reversion – as a contributor to the phenotypic differences observed between the two patients. Mutant transcript levels were similar between homozygotes and heterozygotes, but protein studies in P1 LCLs suggest multiple low-abundance ARPC1B species, including a faint band at the expected size of the wild-type protein. The identity and functional relevance of these products remain uncertain. In contrast, WT ARPC1B transcript corresponded with genotype, with heterozygotes showing intermediate expression and both homozygotes showing reduced levels. P1 demonstrated a higher proportion of retained WT transcript than P2, which may contribute to the milder clinical course, although this relationship cannot be confirmed from the current data and may be influenced by additional genetic or cellular factors.

Despite reduced WT ARPC1B expression, heterozygotes maintained normal F-actin polymerisation, suggesting that a threshold level of ARPC1B protein is sufficient to preserve basal function. The potential role of splice-modulating therapies is of theoretical interest but lies beyond the scope of the current study, and such approaches would require extensive validation before clinical consideration.

Western blotting initially failed to detect ARPC1B in PBMC or PMN lysates from P1, despite the functional data indicating residual activity. Intracellular flow cytometry proved more sensitive, likely reflecting methodological differences: fixation and permeabilisation preserve cellular structure, whereas SDS-PAGE disrupts them through denaturation. This approach demonstrated genotype-dependent clustering of ARPC1 isoform expression and revealed a reciprocal increase in ARPC1A in P1’s LCLs. In P1, residual WT ARPC1B expression was comparable to ARPC1A levels. Interestingly, ARPC1A protein level varied according to ARPC1B genotype, yet *ARPC1A* transcript levels remained comparable across all subjects. This implies that ARPC1A compensation is regulated post-transcriptionally, potentially through altered translation or protein stability, reflecting a cellular strategy to maintain functional integrity in the absence of ARPC1B. However, even the highest ARPC1A levels observed in P1 did not reach the ARPC1B expression seen in healthy donors, reinforcing that ARPC1A cannot fully compensate for ARCP1B deficiency.

A broader CD19 expression profile was observed in the routine peripheral blood immunophenotyping data of the ARPC1B-deficient cases [32]. P2 demonstrated a notably reduced median CD19 expression level compared with P1, which may reflect an increased proportion of less mature B cells. Given that B cell maturation is associated with surface upregulation of CD21 (the ‘EBV receptor’), a relative enrichment of immature B cells could help explain the failure of P2’s B cells to undergo EBV-mediated immortalisation in vitro. Supporting this, actin cytoskeleton remodelling is known to be required for EBV entry into B cells [36].

A limitation of this study was the reliance on retrospective data and limited archived primary material, restricting further investigations. Since both P1 and P2 underwent HSCT, additional primary blood specimens were unavailable. The inability to obtain primary fibroblasts from P2 also prevented further direct comparative analyses of ARPC1B expression and function. Functional differences in neutrophils and B cells were observed, but an opportunity to further perform comparative T and NK cell function assays could not be performed. The intracellular flow cytometric assay, which improved ARPC1B detection over Western blotting, was not available to assess pre-transplant primary blood specimens, preventing verification of ARPC1B protein levels in specific peripheral blood populations. For P2, heterozygous VUS in *LRBA* and *TCN2* were identified. These variants are not associated with disease in the monoallelic state, and further evaluation (including segregation) was not permitted within the diagnostic scope of testing. Although they are unlikely to represent primary causes of P2’s presentation, a potential modifier effect cannot be excluded.

The findings indicate that leaky splicing of ARPC1B^c.64+2T> A^ may contribute to the phenotypic variability observed in this actinopathy. However, this hypothesis requires confirmation in additional patients with the same splice-site variant. More broadly, our observations highlight that variable splice-site usage may represent an under-recognised source of clinical heterogeneity in splicing-related inborn errors of immunity.

## Supplementary Information

Below is the link to the electronic supplementary material.

Supplementary Material 1

## Data Availability

The data supporting the findings of this study are provided within the manuscript and supplementary materials or are available upon reasonable request from the corresponding author.
