# Supplementary material for: Characterisation of a Leaky Splice-Site Mutation Associated with Phenotypic Diversity in Two Unrelated Patients with ARPC1B Deficiency
Source: J Clin Immunol. 2026 Mar 7;46(1):33. doi: 10.1007/s10875-026-02002-4 (PMC13009096; doi:10.1007/s10875-026-02002-4)
Supplement: Supplementary file 1 — Supplementary Material 1 [file 10875_2026_2002_MOESM1_ESM.docx]

**Supplemental Material**

**A leaky splice-site mutation drives phenotypic diversity in two unrelated patients with ARPC1B deficiency**

**Alex Quach^1,2^**

**Jovanka King^1,3^**

**Trishni Putty^1^**

**Michael Gold^3^**

**Patrick Quinn^3^**

**Antonio Ferrante^1,2,4^**

^1^Department of Immunopathology, SA Pathology, Women’s and Children’s Hospital, North Adelaide, Adelaide, South Australia, Australia; ^2^School of Biomedicine, Adelaide Medical School, and the Robinson Research Institute, University of Adelaide, Adelaide, South Australia, Australia; ^3^The Discipline of Paediatrics, School of Medicine, University of Adelaide and Department of Allergy and Clinical Immunology, Women’s and Children’s Health Network, Adelaide, South Australia, Australia; ^4^School of Biological Sciences, University of Adelaide, Adelaide, South Australia, Australia

Corresponding author:

Dr Alex Quach

SA Pathology at the Women’s and Children’s Hospital

Department of Immunopathology, L5 Clarence Reiger Building

72 King William Road, North Adelaide SA 5006

Australia

T: +61 8 8161 6343

E: [alexander.quach@adelaide.edu.au](mailto:alexander.quach@adelaide.edu.au)

ORCID: 0000-0003-1912-7581

**Table S1. Antibodies used in this study**

| **Target** | **Antibody** | **Clone** | **Conjugate** | **Dilution** | **Source** |
| --- | --- | --- | --- | --- | --- |
| ARPC1B | Rabbit anti-ARPC1B*  1. HPA004832 – ARPC1B 225-365aa  2. A302-780A – ARPC1B 272-322aa  3. A302-781A – ARPC1B 322-372aa | Polyclonal | - | 1:1000  1:2000  1:2000 | Sigma-Aldrich  Bethyl Labs  Bethyl Labs |
| ARPC1A | Rabbit anti-ARPC1A (HPA004334) | Polyclonal | - | 1:1000 | Sigma-Aldrich |
| GAPDH | Mouse monoclonal anti-GAPDH | 71.1 | - | 1:40,000 | Sigma-Aldrich |
| Mouse IgG | HRP-conjugated rabbit anti-mouse Ig | - | HRP | 1:2000 | Dako (P0260) |
| Rabbit IgG | HRP-conjugated goat anti-rabbit Ig | - | HRP | 1:2000 | Dako (P0448) |
| CD3 | Mouse anti-CD3 | SK7 | FITC | - | BD Biosciences |
| CD16 | Mouse anti-CD16 | B73.1 | PE | - | BD Biosciences |
| CD56 | Mouse anti-CD56 | MY31 | PE | - | BD Biosciences |
| CD19 | Mouse anti-CD19 | HIB19 | PE-Cy5 | - | BD Biosciences |
| CD4 | Mouse anti-CD4 | RPA-T4 | APC | - | BD Biosciences |
| CD8 | Mouse anti-CD8 | RPA-T8 | PE-Cy7 | - | BD Biosciences |
| CD45 | Mouse anti-CD45 | 2D1 | APC-H7 | - | BD Biosciences |
| CD11b | Mouse anti-CD11b | D12 | PE | - | BD Biosciences |
| Isotype Control | Mouse IgG1-κ | MOPC-21 | PE | - | BD Biosciences |
| CRIg/Z39Ig | Mouse anti-Z39Ig | 6H8 | PE | - | Santa Cruz Biotechnology |

Dilution refers to usage in Western blotting.

*Note that three polyclonal antibodies to ARPC1B were used in this study – the catalogue numbers and immunogen amino acid (aa) sequence intervals are listed.

**Table S2. Sequences of oligonucleotides used for qPCR of ARPC1B wildtype and c.64+2T>A mutant transcripts.**

| **Design** | |  |  |  |
| --- | --- | --- | --- | --- |
| **#** | **Name** | **Type** | **Direction** | **Sequence** |
| 1 | ARPC1B Ex 2 WT F | Amplification | F | CATGGCCTACCACAGCTTCC |
| 2 | ARPC1B Ex 2-3 WT BLK R | Blocking | R | GATGGCAATCTGGGTGCG-3’PO_4_ |
| 3 | ARPC1B Ex 2-3 WT R | Amplification | R | CAGATGGCAATCTGGGTGC |
| 4 | ARPC1B Ex 2 Mut BLK R | Blocking | R | AAGCACTCTCGGGTGCG-3’PO_4_ |
| 5 | ARPC1B Ex 2-3 Mut R | Amplification | R | CAGATGGCAATCTCGGCC |
| 6 | ARPC1A Ex 2-3 mRNA F | Amplification | F | AACAGGGATCGTACTCAGATTG |
| 7 | ARPC1A Ex 3 mRNA R | Amplification | R | CTGGCTCCCGTTCTTCTTATAG |
| 8 | GAPDH mRNA F | Amplification | F | GAGTCAACGGATTTGGTCGT |
| 9 | GAPDH mRNA R | Amplification | R | GACAAGCTTCCCGTTCTCAGCCT |
| **Primer combinations** | |  |  |  |
| **Target** | | **Combination** | **Notes** | |
| ARPC1B WT allele | | # 1, 3, 4 | WT exon 2-3 junction is targeted by reverse amplification primer. Blocking primer targets mutant exon 2-3 junction. | |
| ARPC1B c.64+2T>A allele | | # 1, 2, 5 | Mutant exon 2-3 junction is targeted by reverse amplification primer. Blocking primer targets WT exon 2-3 junction. | |
| ARPC1A | | # 6, 7 | Exon 2-3 junction is targeted by forward amplification primer. | |
| GAPDH | | # 8, 9 | Housekeeping gene for normalisation. | |

Primers were designed using a similar strategy to that described by Alvarez-Garcia et al. [1]: adapted from detection of SNV in gDNA to detection of alternate transcripts due to splice-site mutation and activation of cryptic splice-site in cDNA transcribed from total RNA. The positions of the ARPC1B-specific primer sequences are represented in Fig. 6A.

**Table S3. ARPC1B mutations reported to date.**

| **NM_005720.4**  **c.** | **NP_005711.1**  **p.** | **Variant type** | **Ethnicity** | **No. of**  **kindreds** | **References** |
| --- | --- | --- | --- | --- | --- |
| c.64C>T  (Compound heterozygous with c.899_944del) | p.Q22* | Nonsense | Mexican | 1 | Vasquez-Echeverri et al. [2] |
| c.64+1G>A | p.T21insPECLLGA | Splice-site | Turkish | 1 | Brigida et al. [3] |
| c.64+1G>C | p.T21insPECLLGA | Splice-site | Italian | 1 | Brigida et al. [3] |
| c.64+2T>A **(*)** | p.? | Splice-site | Nepalese | 13 | Volpi et al. [4]  Bhattarai et al. [5] |
| c.94G>A  (Compound heterozygous with c.111G>C) | p.V32M | Missense | Mexican | 1 | Vasquez-Echeverri et al. [2] |
| c.111G>C  (Compound heterozygous with c.94G>A) | p.K37N | Missense | Mexican | 1 | Vasquez-Echeverri et al. [2] |
| c.212_226del | p.G71_N75del | In-frame deletion | Italian | 1 | Chiriaco et al. [6] |
| c.258G>A | p.W86* | Nonsense | Columbian | 1 | Brigida et al. [3] |
| c.265A>C | p.T89P | Missense | Slovenian | 4 | Kopitar et al. [7] |
| c.269_270dup | p.V91Wfs*30 | Duplication/frameshift | South Asian | 1 | Kahr et al. [8]  Leung et al. [9] |
| c.311G>C | p.W104S | Missense | Moroccan | 1 | Volpi et al. [4] |
| c.314C>T  (inherited with c.712G>A, p.A238T presumed neutral) | p.A105V | Missense | Scottish | 1 | Kahr et al. [8]  Leung et al. [9] |
| c.318del | p.N107Tfs*13 | Deletion/frameshift | Moroccan | 1 | Brigida et al. [3] |
| c.392+2T>C | p.? | Splice-site | Somalian | 1 | Volpi et al. [4] |
| c.491_495delinsCCTGCCC | p.F164Sfs*32 | Indel/frameshift | Moroccan | 1 | Kuijpers et al. [10] |
| c.622G>T | p.V208F | Missense | Italian | 1 | Brigida et al. [3] |
| c.623_624del | p.C209Ffs*21 | Deletion/frameshift | Israeli | 1 | Somech et al. [11] |
| c.708-1G>A | p.? | Splice-site | Jordanian | 1 | Volpi et al. [4] |
| c.783G>A  (last nucleotide of exon 7) | p.A261= | Splice-site | Afghan | 1 | Papadatou et al. [12] |
| c.863del | p.P288Lfs*9 | Deletion/frameshift | Mexican | 1 | Zaveleta Martinez et al. [13] |
| c.897_910del | p.E300Pfs*153 | Deletion/frameshift | Iranian | 2 | Volpi et al. [4] |
| c.899_944del | p.E300Gfs*7 | Deletion/frameshift | Mexican | 1 | Castano-Jaramillo et al. [14]  Vasquez-Echeverri et al. [2]  Zaveleta Martinez et al. [13] |
| c.1081-5T>G | p.? | Splice-site | Turkish | 1 | Sonmez et al. [15] |
| c.1087dup | p.E363Gfs*95 | Duplication/frameshift | Canadian | 1 | Brigida et al. [3] |
| Unknown | - | Splice-site | Kenyan | 1 | Antala et al. [16] |

(*), the subject of this study.

**Table S4. Additional laboratory investigations.**

| **Measurand** | **Reference**  **range** | **Units** | **P1** | **P1 HD** | **P2** | **P2 HD** |
| --- | --- | --- | --- | --- | --- | --- |
| Dihydrorhodamine-123  oxidase burst assay | > 35 | Neutrophil  Oxidative Index | 61.9  (6 years) | 83.1 | 252.5  (3.5 months) | 83.4 |
| T cell receptor excision circles (TRECs) | > 8165 | TRECs/µg lymphocyte DNA | ND | - | 36539  (3.5 months) | - |
| Lymphocyte proliferation  Phytohaemagglutinin (PHA) | 17.2 – 618.1 | Stimulation Index  (SI) | Baseline DPM: 3644.4  PHA DPM: 204644.6  SI: 56.2 (11 months) | Baseline DPM: 1508.1  PHA DPM: 84245.8  SI: 55.9 | Baseline DPM: 4057.3  PHA DPM: 124635.8  SI: 30.7 (4.5 months) | Baseline DPM: 682.8  PHA DPM: 155715.0  SI: 228.1 |
| Lymphocyte proliferation  Pokeweed mitogen (PWM) | 8.9 – 301.4 | Stimulation Index  (SI) | DPM: 40955.8  SI: 11.2 (11 months) | DPM: 41970.7  SI: 27.8 | ND | - |
| Lymphocyte proliferation  Concanavalin A (ConA) | 14.8 – 593.2 | Stimulation Index  (SI) | DPM: 88459.6  SI: 24.3 (11 months) | DPM: 63359.8  SI: 42.0 | ND | - |
| Lymphocyte proliferation  *Staphylococcus aureus* | 1.4 – 42.2 | Stimulation Index  (SI) | DPM: 17696.7  SI: 4.9 (11 months) | DPM: 4753.9  SI: 3.2 | ND | - |
| Lymphocyte proliferation  Anti-CD3 | > 2.3 | Stimulation Index  (SI) | ND | - | DPM: 27645.8  SI: 6.8 (4.5 months) | DPM: 50206.6  SI: 73.5 |
| Lymphocyte proliferation  Anti-CD3 + Anti-CD28 | > 11.2 | Stimulation Index  (SI) | ND | - | DPM: 209847.9  SI: 51.7 (4.5 months) | DPM: 195845.2  SI: 286.8 |
| IgG_1_ | 2.07 – 6.69  2.48 – 7.31  3.86 – 9.66 | g/L  g/L  g/L | 11.37 *H (11 months)  6.95 *H (17 months)  8.84 *H (24 months)  10.57 *H (25 months)  15.04 *H (5 years) | - | ND | - |
| IgG_2_ | 0.38 – 2.05  0.47 – 2.44  1.19 – 5.4 | g/L  g/L  g/L | 2.52 *H (11 months)  2.21 *H (17 months)  1.80 (24 months)  2.35 (25 months)  1.76 (5 years) | - | ND | - |
| IgG_3_ | 0.09 – 0.52  0.10 – 0.57  0.16 – 0.75 | g/L  g/L  g/L | 0.48 (11 months)  0.39 (17 months)  0.62 *H (24 months)  0.73 *H (25 months)  1.28 *H (5 years) | - | ND | - |
| IgG_4_ | 0.01 – 0.36  0.018 – 0.49  0.035 – 0.66 | g/L  g/L  g/L | 0.45 *H (11 months)  0.40 *H (17 months)  0.45 *H (24 months)  0.42 (25 months)  0.003 *L (5 years) | - | ND | - |
| C3 | 0.74 – 1.38  0.8 – 1.5 | g/L  g/L | -  1.18 (24 months)  1.23 (6.5 years) | - | 1.02 (3 months) | - |
| C4 | 0.12 – 0.36 | g/L | 0.11 *BL (24 months)  0.15 (6.5 years) | - | 0.10 *BL (3 months) | - |
| CH50 | 59 – 132 | % | 85 (11 months) | - | 128 (4 months) | - |

The approximate age of P1/P2 at the time of testing is indicated in brackets adjacent the result. The corresponding healthy donor (P1/P2 HD) control result is included for relevant cellular function testing. For lymphocyte proliferation (^3^H-thymidine uptake) assays, the baseline/control disintegrations per minute (DPM) was used to generate stimulation indices. ND, not determined; *H, high; *L, low; *BL, borderline.


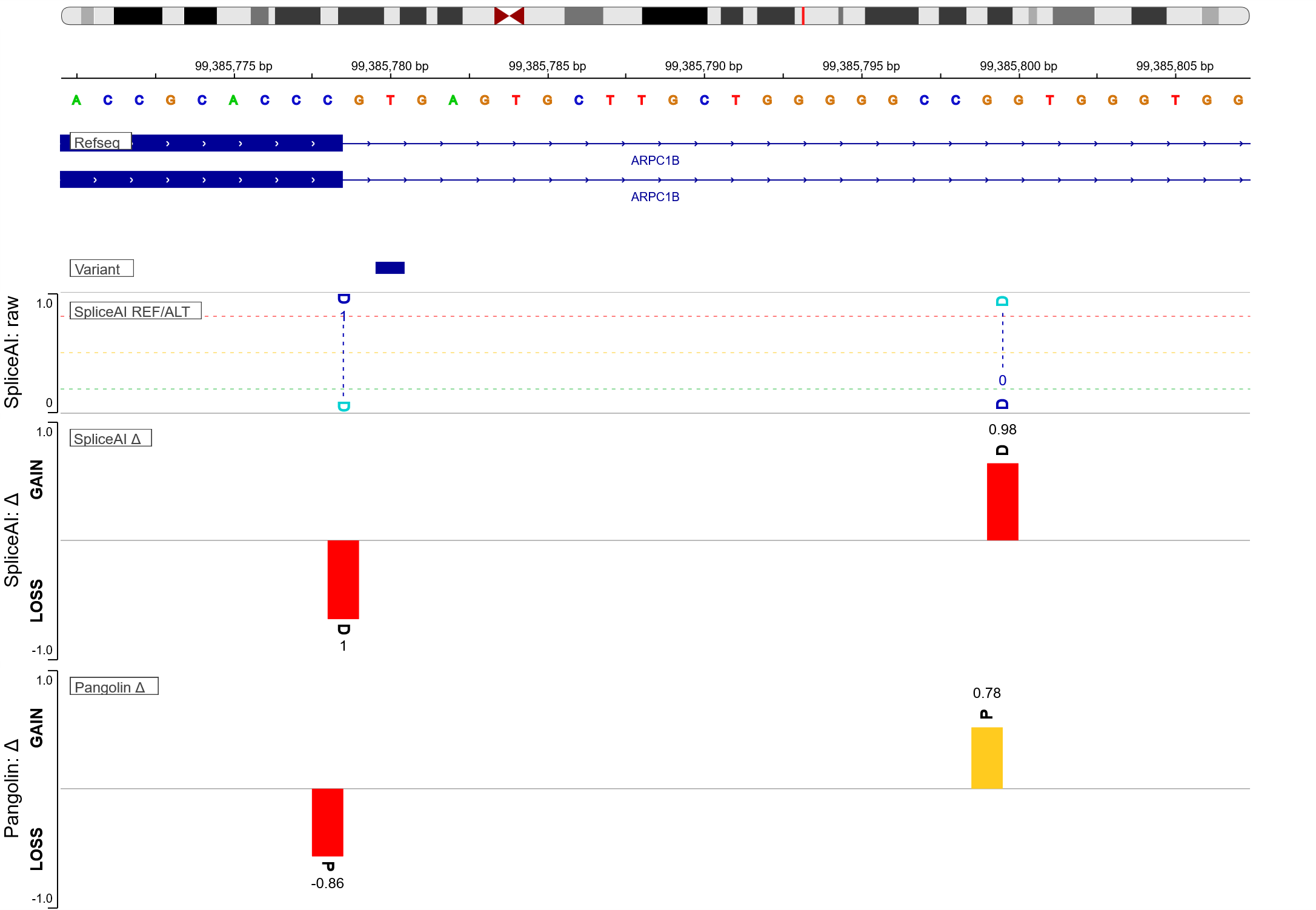


**Figure S1. SpliceAI and Pangolin prediction scores for RNA splicing by the** **ARPC1B^c.64+2T>A^ substitution.** SpliceAI and Pangolin are deep learning models for RNA splicing prediction, generating scores ranging from -1 to 1 to indicate a loss or gain of donor/acceptor splice sites. Both predict ARPC1B^c.64+2T>A^ to cause a ‘loss’ of the original donor site (-2 bp from the substitution site), and a ‘gain’ in a donor capacity of the downstream cryptic splice-site (+19 bp from the substitution site). Scores were accessed from spliceailookup.broadinstitute.org on 07 March 2025.


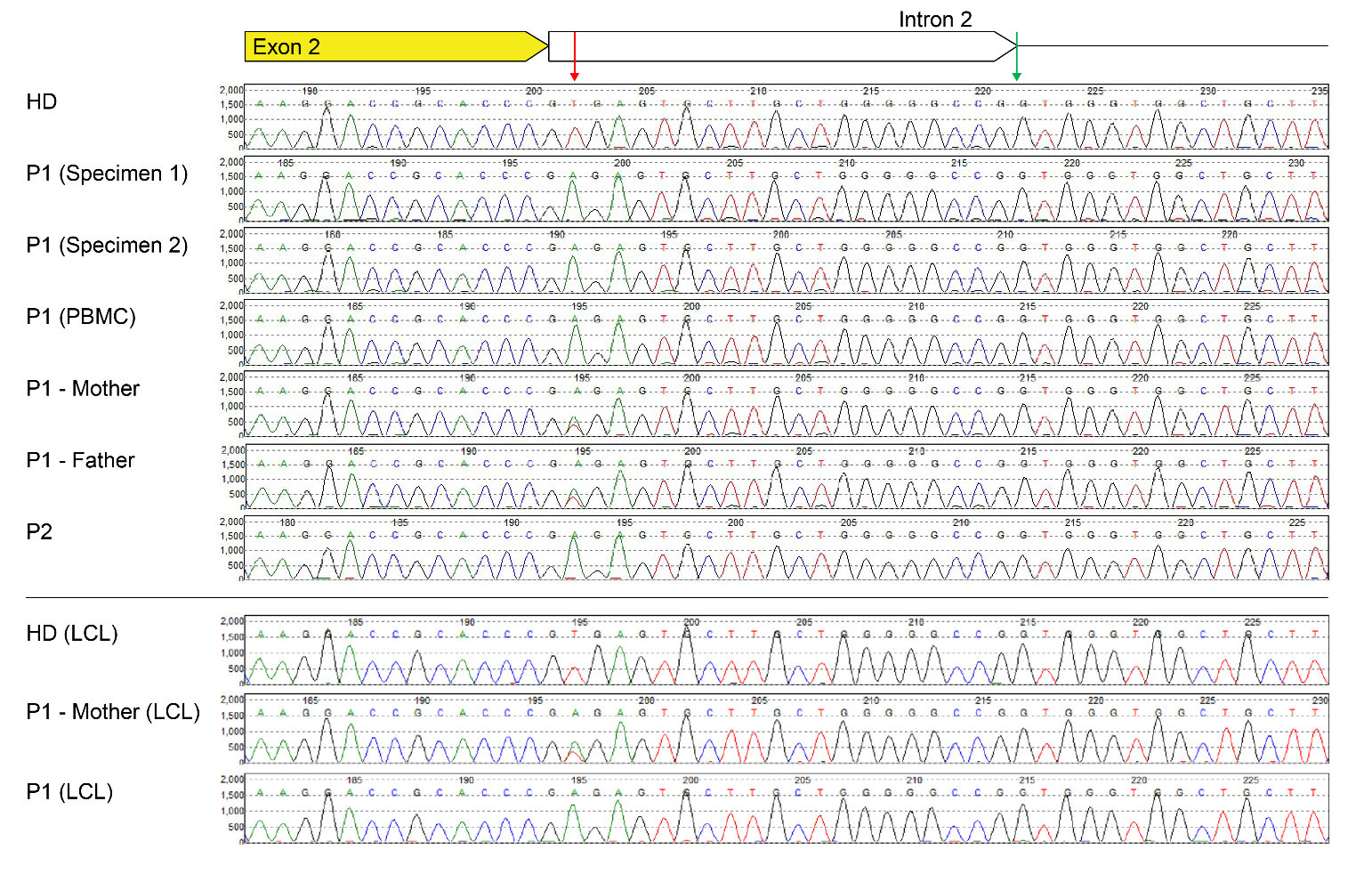


**Figure S2. Genomic sequencing of the ARPC1B^c.64+2T>A^ site.** Chromatograms from the indicated individuals are aligned. Whole blood was the source of the genomic DNA unless otherwise indicated. The yellow bar indicates ARPC1B exon 2, whilst the white bar indicates the intronic 21 bp sequence included in the mutant transcript. The red arrow indicates the affected base, whilst the green arrow indicates the cryptic splice-site that is activated by the mutation.

**Supplemental References**

1. Alvarez-Garcia V, Bartos C, Keraite I, Trivedi U, Brennan PM, Kersaudy-Kerhoas M, et al. A simple and robust real-time qPCR method for the detection of PIK3CA mutations. Sci Rep. 2018;8(1):4290.

2. Vasquez-Echeverri E, Yamazaki-Nakashimada MA, Venegas Montoya E, Scheffler Mendoza SC, Castano-Jaramillo LM, Medina-Torres EA, et al. Is Your Kid Actin Out? A Series of Six Patients With Inherited Actin-Related Protein 2/3 Complex Subunit 1B Deficiency and Review of the Literature. J Allergy Clin Immunol Pract. 2023;11(4):1261-80 e8.

3. Brigida I, Zoccolillo M, Cicalese MP, Pfajfer L, Barzaghi F, Scala S, et al. T-cell defects in patients with ARPC1B germline mutations account for combined immunodeficiency. Blood. 2018;132(22):2362-74.

4. Volpi S, Cicalese MP, Tuijnenburg P, Tool ATJ, Cuadrado E, Abu-Halaweh M, et al. A combined immunodeficiency with severe infections, inflammation, and allergy caused by ARPC1B deficiency. J Allergy Clin Immunol. 2019;143(6):2296-9.

5. Bhattarai D, Banday AZ, Patra PK, Baral R, Chaudhry C, Girisha KM, et al. The c.64 + 2 T > A Founder Variant Hits Home: Report on 14 Patients Expands the Phenotypic Landscape of Inherited ARPC1B Deficiency - a Comparative Analysis. Clin Rev Allergy Immunol. 2025;68(1):64.

6. Chiriaco M, Ursu GM, Amodio D, Cotugno N, Volpi S, Berardinelli F, et al. Radiosensitivity in patients affected by ARPC1B deficiency: a new disease trait? Front Immunol. 2022;13:919237.

7. Kopitar AN, Markelj G, Orazem M, Blazina S, Avcin T, Ihan A, et al. Flow Cytometric Determination of Actin Polymerization in Peripheral Blood Leukocytes Effectively Discriminate Patients With Homozygous Mutation in ARPC1B From Asymptomatic Carriers and Normal Controls. Front Immunol. 2019;10:1632.

8. Kahr WH, Pluthero FG, Elkadri A, Warner N, Drobac M, Chen CH, et al. Loss of the Arp2/3 complex component ARPC1B causes platelet abnormalities and predisposes to inflammatory disease. Nat Commun. 2017;8:14816.

9. Leung G, Zhou Y, Ostrowski P, Mylvaganam S, Boroumand P, Mulder DJ, et al. ARPC1B binds WASP to control actin polymerization and curtail tonic signaling in B cells. JCI Insight. 2021;6(23).

10. Kuijpers TW, Tool ATJ, van der Bijl I, de Boer M, van Houdt M, de Cuyper IM, et al. Combined immunodeficiency with severe inflammation and allergy caused by ARPC1B deficiency. J Allergy Clin Immunol. 2017;140(1):273-7 e10.

11. Somech R, Lev A, Lee YN, Simon AJ, Barel O, Schiby G, et al. Disruption of Thrombocyte and T Lymphocyte Development by a Mutation in ARPC1B. J Immunol. 2017;199(12):4036-45.

12. Papadatou I, Marinakis N, Botsa E, Tzanoudaki M, Kanariou M, Orfanou I, et al. Case Report: A Novel Synonymous ARPC1B Gene Mutation Causes a Syndrome of Combined Immunodeficiency, Asthma, and Allergy With Significant Intrafamilial Clinical Heterogeneity. Front Immunol. 2021;12:634313.

13. Zaveleta Martinez O, Fregoso-Zuniga AE, Razo Requena C, Espinosa Padilla S, Blancas Galicia L. Description of a Novel Pathogenic Variant in the ARPC1B and a Severe Allergy in Two Infants. Iran J Allergy Asthma Immunol. 2024;23(1):122-6.

14. Castano-Jaramillo LM, Yamazaki-Nakashimada MA, Scheffler Mendoza SC, Bustamante-Ogando JC, Espinosa-Padilla SE, Lugo Reyes SO. A male infant with COVID-19 in the context of ARPC1B deficiency. Pediatr Allergy Immunol. 2021;32(1):199-201.

15. Sonmez G, Ulum B, Tenekeci AK, Caka C, Sahin A, Kazancioglu A, et al. Recurrent eosinophilia with a novel homozygous ARPC1B mutation. Front Med. 2024.

16. Antala S, Whitehead B, Melin-Aldana H, Bass LM. ARPC1B Mutation Manifesting as Recurrent Hematemesis With Metaplasia. JPGN Rep. 2021;2(3):e095.
